# Supplementary figures and images for: Pitfalls associated with evaluating enzymatic quorum quenching activity: the case of MomL and its effect on Pseudomonas aeruginosa and Acinetobacter baumannii biofilms
Source: PeerJ. 2017 Apr 27;5:e3251. doi: 10.7717/peerj.3251 (PMC5410158; doi:10.7717/peerj.3251)

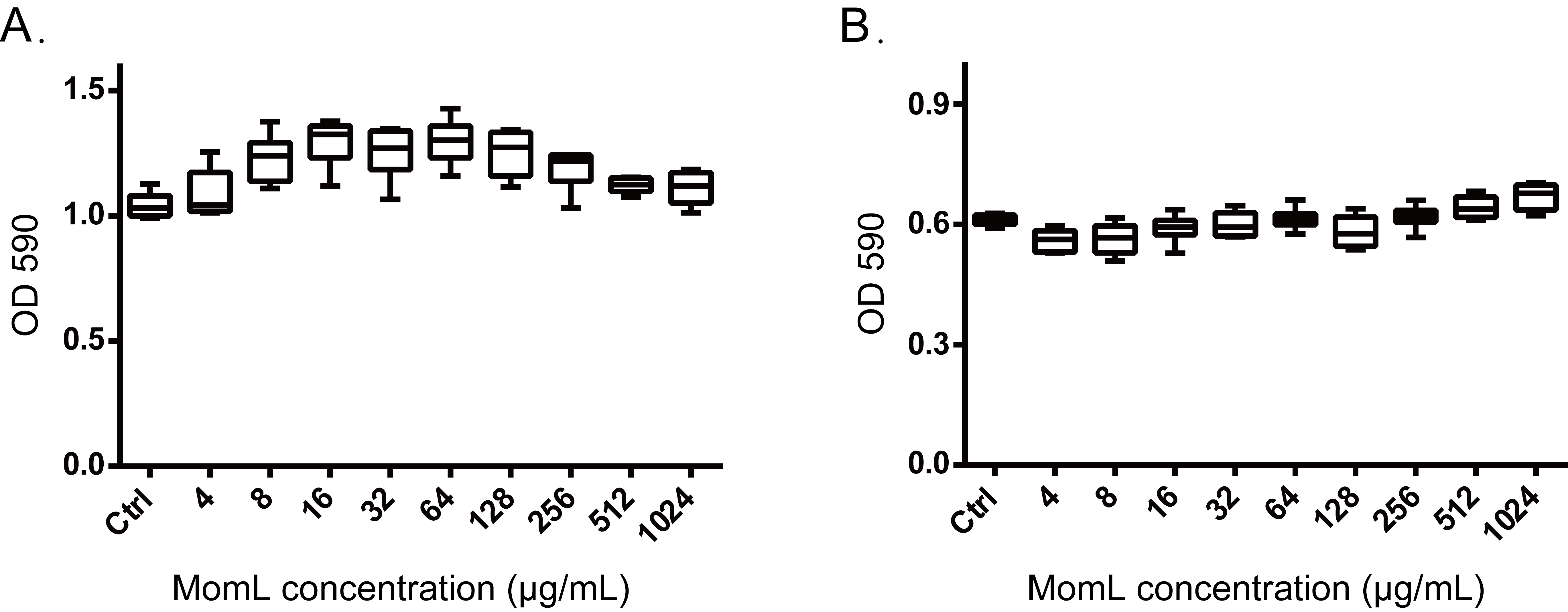

Supplement: Figure S1 — The growth of P. aeruginosa PAO1 and A. baumannii LMG 10531 was spectrophotometrically determined at OD 590 after being incubated with different concentrations of MomL at 37 °C for 24 h. No MomL was added in control. Data shown in box-whisker plots are from three biological replicates with two technical replicates each (n = 6). Boxes span the interquartile range; the line within each box denotes the median, and whiskers indicate the minimum and maximum values. [file peerj-05-3251-s001.png]

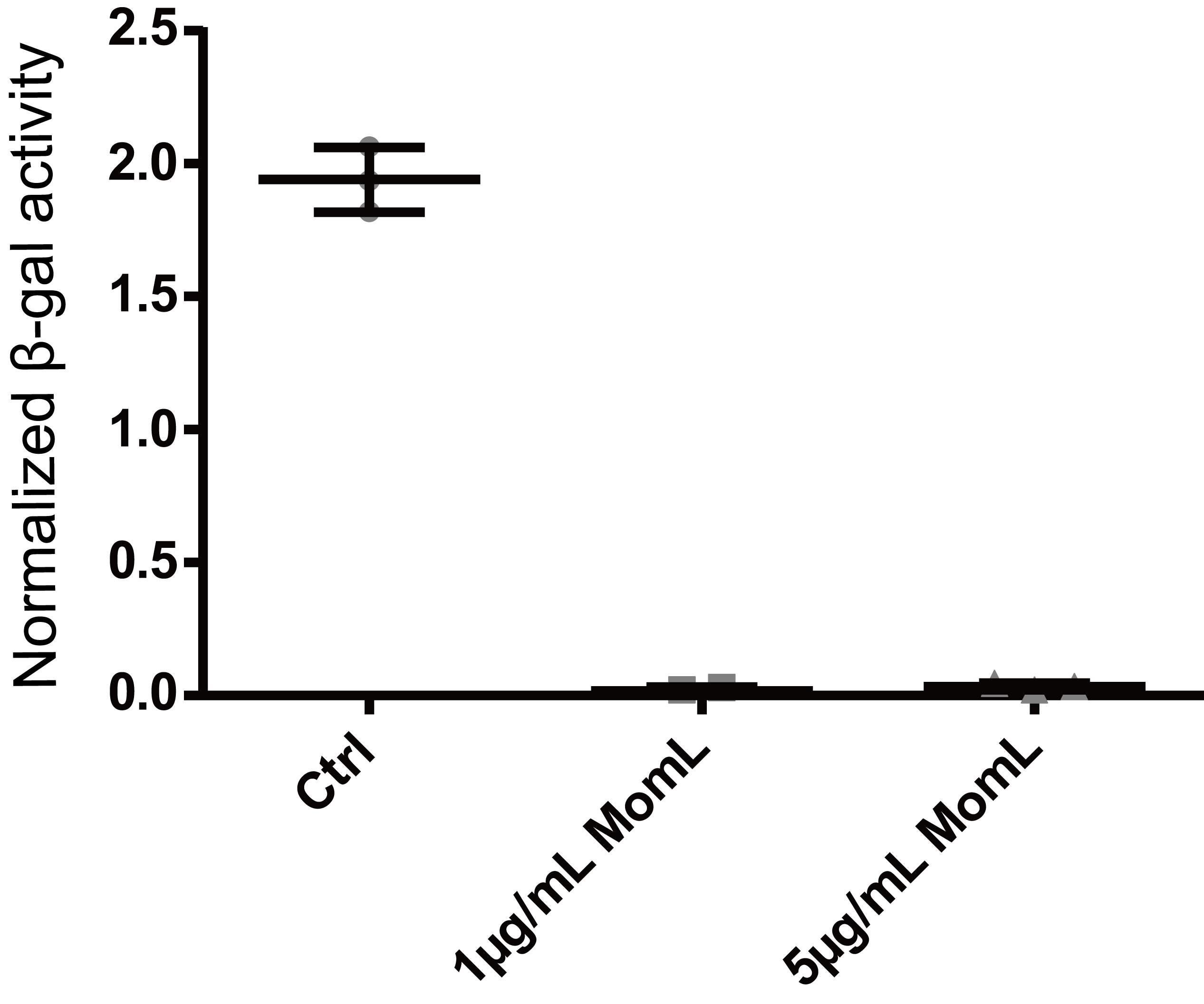

Supplement: Figure S2 — 3-OH-C12-HSL (10 µM) was mixed with MomL under the same medium condition in C. elegans model and incubated at 37°C for 1h. No MomL was added in control. The amount of residual 3-OH-C12-HSL was quantified using A. tumefaciens A136 liquid X-gal assay and expressed as the normalized β-galactosidase activity. [file peerj-05-3251-s002.png]
